# The effect of knowledge transfer theory on the selection of crossover winter sports athletes: A systematic literature review

**DOI:** 10.3389/fpsyg.2022.1001082

**Published:** 2023-02-06

**Authors:** Yongqi Gao, Kim Geok Soh, Noor Syamilah Zakaria, Roziah Mohd Rasdi, Wei Guo, Kim Lam Soh

**Affiliations:** ^1^Department of Sports Studies, Faculty of Educational Studies, Universiti Putra Malaysia, Seri Kembangan, Malaysia; ^2^Department of Counselor Education and Counseling Psychology, Faculty of Educational Studies, Universiti Putra Malaysia, Seri Kembangan, Malaysia; ^3^Department of Professional Development and Continuing Education, Faculty of Educational Studies, Universiti Putra Malaysia, Seri Kembangan, Malaysia; ^4^Department of Nursing, Faculty of Medicine and Health Sciences, Universiti Putra Malaysia, Seri Kembangan, Malaysia

**Keywords:** knowledge transfer, crossover athletes selection, winter sports, knowledge, talent transfer, talent identification

## Abstract

This article reviews studies on the knowledge transfer theory (KTT) in sport psychology using the Preferred Reporting Items for Systematic reviews and Meta-Analyses (PRISMA) method to identify any existing research gaps. This review utilizes a systematic process that involves searching for studies aimed at clarifying the relationship between KTT and crossover selection, promoting crossover development of winter sports, and cultivating outstanding athletes across six different databases. This work provides the foundation for future research on KTT in the field of selection of athletes for professional sports and those intending to showcase KTT’s success in the selection of winter sports athletes. This review found that crossover selection of qualified athletes helps solve the issue of the shortage of professional athletes in specific sports.

## Introduction

The Winter Olympics can demonstrate the image of the country and promote the development of new sports (Hao, 2020; Yixin, 2022). On the one hand, in recent years, the battle between countries to win medals at major sports events, such as the Olympic Games, has intensified (Vaeyens et al., 2009). This may be proxied by the increased investments in competitive sports by the competing countries (Yanli, 2020; Yang, 2021). For example, the value of investments made in the attempt to win an Olympic gold medal in Australia has been estimated to be around 37 million dollars (Hogan and Norton, 2000; Australian Sports Commission, 2009). On the other hand, in tandem with this increasing trend in investments, an increasing number of new sports have surfaced as a result of the integration of sports (Wu, 2020). Take the Winter Olympics events as an example. Freestyle skiing is the result of the combination of alpine skiing, gymnastics, and skill sports. Short-track speed skating is the result of the development of North American athletics. Furthermore, long-track, short-track speed skating, and short-track speed skating are the results of the extension of cycling. Moreover, ski jumping has been extended to grass. On top of that, crossover athletes for cross-country skiing are from track and field, cycling, rowing, and roller skating. Meanwhile, rock climbing, surfing, skateboarding, and small-wheeled vehicles are the other four source sports (i.e., for other minor competitions) in the Winter Olympics (Maijiu et al., 2018).

When selecting athletes for Olympic events, many countries use the crossover selection method (CSM), which is a specific athlete selection (i.e., crossover athletes) procedure that is a part of the theoretical system of athlete selection. The CSM is a formalized process of identifying and developing talented athletes by selecting individuals who have already succeeded in one sport and transferring them to another (Collins et al., 2014). The term “crossover” refers to athletes who make the transition from one sport to the elite level of another (Vaeyens et al., 2009). The CSM has the advantage of being able to identify the factors that may influence the successful transfer of athletes, effectively facilitating talent transfer (MacNamara and Collins, 2015).

There are also other definitions of the CSM. According to Qunru (2019), the CSM is a process in which athletes move from one sport to another—Where the social background, purpose, and selection of materials are similar—To achieve rapid growth. Meanwhile, Collins et al. (2014) stated that, by convincing already matured, experienced, and talented performers to try a new sport, the CSM allows the athletes to develop their skills to become equally (or even more) successful in the new pursuit. Moreover, MacNamara and Collins (2015) described CSM as the initiative that seeks to transfer talented, mature individuals from one sport to another. It refers to the mode in which athletes who have a certain sports foundation or have achieved success in a certain sport seek development by switching to other sports (Vaeyens et al., 2009). Collins et al. (2014) investigated the influence on other athletes of screening the selection of crossover winter sports athletes who have been successful in a certain sport.

Furthermore, knowledge transfer theory is defined as an athlete’s learning in one sport that affects his learning in other sports (Farrow et al., 2013). This theory focuses on the influence of the original sport’s knowledge structure on the learning of new sports (Guoquan, 1989). KTT provides a theoretical reference for the selection of athletes (Qunru, 2019). It serves as a theoretical cornerstone for crossover selection, which drives the elements that affect the selection of crossover athletes (Di and Xu, 2016). Additionally, within the field of research and in practice, KTT may assist in developing a comprehensive and well-integrated talent development system (Taufiq et al., 2021).

According to the statistics on the development trends of winter sports, China participated in seven major events in the 2014 Sochi Winter Olympics. Although the poor foundation of winter sports has improved, and its popularity rate has increased, there is still a lack of studies on industry trends (Wernerfelt, 1984). There is also a gap between the existing number of athletes and those required in international winter sports games, where the population of athletes does not meet the actual demand (Yumei et al., 2006). Given the current situation and the need for crossover athletes’ selection in winter sports, the integration of KTT in crossover selection and its application as discussed in this review is urgently needed.

There are only three provinces in China that are particularly favorable for winter sports development due to their natural environments (Heilongjiang, Jilin, and Liaoning). Therefore, there is uneven development in winter sports. These provinces are all located in the Northeast region of the country. By 2020, the number of winter sites in Northeast China accounted for two-thirds of the country’s winter sports facilities and key projects. Meanwhile, the development of winter sports in these provinces has been greatly affected as athletes’ training outside the winter season is restricted.

As winter sports are subject to seasonal constraints, these types of sports prioritize competition over universality, resulting in a small number of winter clubs being opened to the public with a few participants (Song et al., 2020). Winter sports depend on seasonal factors during the cold seasons. Therefore, traditionally, outdoor weather conditions often affect the public’s motivation to participate in winter sports. In addition, the cost of winter sports is relatively high in terms of equipment and venues. Accordingly, the public usually prefers to opt for more affordable sports over winter sports due to cost concerns (Sun and Gong, 2019).

In the 19th, 20th, 21st, 22nd, and 23rd Winter Olympics, China won the 13th, 14th, 8th, 12th, and 16th place in the World Medal List, respectively. Nevertheless, China’s performance in the Winter Olympics was inconsistent with a less substantial competitive advantage over other countries. China’s award-winning sports are mainly short-track speed skating, figure skating, and other ice-skating events. Short-track speed skating has gradually become a popular event, where Chinese athletes with stellar skills often dominate the competitive stage (Wang et al., 2019; Wu, 2020).

Furthermore, the number of participants in winter events in China is relatively small. This is even worse for the less popular winter sports due to geographical limitations such as solid ice and weak snow (Tai et al., 2020). Based on the above discussion, it may be inferred that winter sports are still underdeveloped in China. This review explores multiple solutions to address this problem based on a theoretical framework that emphasizes crossover selection. This review is based on the initial assumption that constructing a comprehensive professional winter athletes training model will have a profound effect on the development of winter sports in China.

## Materials and methods

### Protocol and registration

**FIGURE 1:**
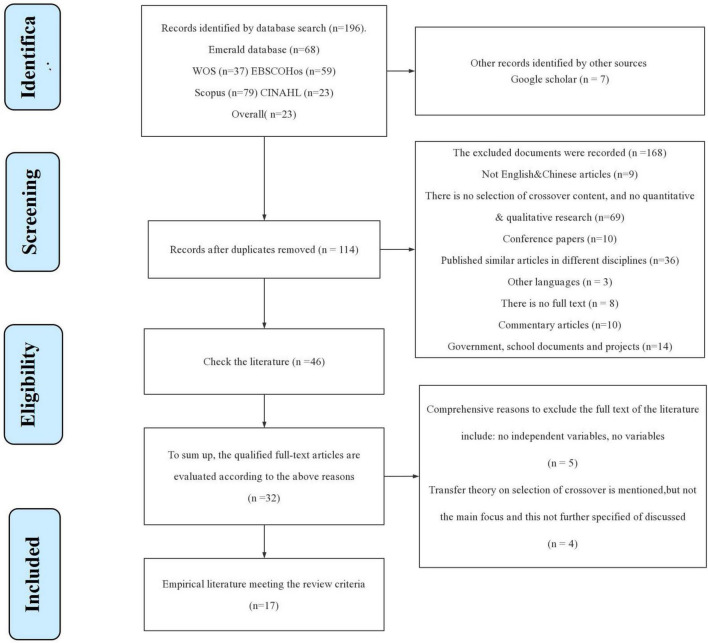
Preferred Reporting Items for Systematic reviews and Meta-Analyses flowchart for systematic literature review.

This review adopts the PRISMA guideline, which is depicted in Figure 1. This guideline includes the process of inclusion and exclusion of studies based on certain set criteria (Moher et al., 2009). On top of that, the researcher was also prospectively registered on the International Platform of Registered Systematic Review and Meta-analysis Protocols, with the details summarized in Table 1.

**TABLE 1:** Protocol registration information.

Item	Details
URL	https://inplasy.com/
Registration number	INPLASY202260090
DOI number	10.37766/inplasy2022.6.0090

### Research characterization and data analysis procedure

Figure 1 shows the process of evaluating the suitability of results from the initial search to be included for further review. PRISMA systematically reviews the flow of information at various stages based on the KTT and crossover athletes’ selection (Moher et al., 2009). Specifically, there were four steps undertaken in the selection of research results for review, including (1) identification, (2) screening, (3) eligibility, and (4) inclusion. Finally, the Population, Intervention, Comparison, Outcomes, and Study (PICOS) tool (Ohrt et al., 2020) was utilized to present some selected research content.

### Search strategy

The relevant studies were retrieved from six databases, namely, (i) Emerald, (ii) Web of Science, (iii) EBSCOHOS, (iv) SCOPUS, (v) CNKI, and (vi) CINAHL. This breadth of coverage allows for a comprehensive review of the latest, historical, specific, and multidisciplinary research outcomes. Keywords and titles were chosen as the main searching methods, and the TITLE-ABS-KEY were “an empirical examination” OR “winter sports” OR “knowledge transfer theory” OR “athletes learning” OR “selecting and cultivating athletes” OR “crossover sports” OR “winter Olympics” OR “crossover sports theory” OR “crossover sports and projects” OR “athletes’ options” OR “china winter sports” OR “crossover winter sports” OR “knowledge transfers” OR “knowledge embeddedness” OR “successful knowledge transfers” OR “transfer theory” OR “talent identification.”

### Inclusion criteria and search results

Based on the above search criteria, the literature search covers data between the years 2003 and 2021; 196 articles were shortlisted from the initial round of search, with 114 of them excluded for the following reasons: (1) Not a research study, (2) no factors related to KTT, and (3) involved population who are not sports athletes. An in-depth review was conducted of the filtered 19 articles, with particular attention given to the group under study. An all-around cross-check on the relevance of the included articles was performed to ensure that they meet the inclusion criteria (Jesson et al., 2011; Heisig and Kannan, 2020). We identified 17 research conducted involving intervention studies that investigate the effect of KTT on the selection of crossover winter sports athletes.

The criteria for exclusion include (1) articles not in English or Chinese, (2) absence of selection of crossover-related content, (3) absence of quantitative and qualitative research, (4) articles not in full text, and (5) commentary articles that are not peer-reviewed.

### Preliminary analysis of articles to uncover the KTT criteria

A preliminary analysis of the articles was conducted to uncover the KTT Criteria. The integrated theory of KTT and crossover athletes’ selection mainly include concepts such as “Talent transfer (TT),” “TT program,” “TT initiatives,” and “Mature-age talent and talent recycling program.” In the context of China, relevant concepts that repeatedly surfaced in the literature include “athletes’ sports games conversion” and “crossover athletes’ success.” Most of the research was conducted on the selection of crossover athletes *via* various projects, where its theory and methods were proven effective. Some research also suggests that the characteristics of crossover skiers’ bodies (of different genders and sports events) provide a reference value for selecting other crossover skiers. In addition, some research also suggests that KTT enables an objective selection of athletes that facilitates the transfer of content knowledge from one sport to another (Ward et al., 2015; Rilang, 2020).

### Final considerations

Although crossover athlete selection is getting more mature in the field of pedagogical psychology related to transfer theory (Murawski and Lee Swanson, 2001), there is still a lack of, or even a gap in, content knowledge transfer research in the field of physical education. This review aims to close the gaps found in the existing research in this field. It is emphasized that the success of content knowledge transfer needs to be proven in actual practice. The likelihood of content knowledge transfer is enhanced with participation in various practical activities (Nair et al., 2021). Moreover, the greater the transfer, the greater the receivers’ ability to internalize content knowledge (Jeffrey, 2003; van Harten et al., 2021).

### Knowledge transfer theory outcomes

The research of KTT originated from Al-Fedaghi (2012) mathematical theory of communication (Hongchang et al., 2006). The subsequent development is the joint work by economists (Arrow, 1969) and sociologists (Rogers, 1962). The early theory was mainly divided into formal discipline theory, standard element theory, transposition theory, and generalization theory (Cummings and Teng, 2003).

Studies on crossover sports under KTT show that the implicit and explicit effects of content knowledge transfer are positive (Allen et al., 1979; Berry and Broadbent, 1987; Starbuck, 1992). KTT leads to the receiver’s accumulation or absorption of new content knowledge. Pavlenko and Jarvis (2002) were the first researchers who utilized the concept of transfer and formulated their theory to test the conceptual transfer hypothesis. At that time, the study of KTT was only on the surface and mainly related to the internal structure of language and conceptual categories. Subsequently, Murphy (2002) categorized it into a homogeneous group based on its essential properties. On the contrary, Li (2006) explained the transfer principle based on a dynamic and multidimensional theoretical framework.

A transfer is defined as a certain kind of similarity between two things (Tian et al., 2018). Knowledge transfer is a fundamental part of the learning process and belongs to the knowledge category called “psychology.” In addition, “transfer effect” refers to the impact of previously learned skills on subsequently undertaken sports. This effect is due to, among other things, the homogeneous factors present between the two sports. Moreover, KTT refers to the effect of one type of learning on another, either positive or negative (or sometimes called disturbances). This process of transfer is widespread in content knowledge, behaviors, and skills. By exploring this theme, this review focuses on the transfer and advancement between previous and successive learning.

Furthermore, KTT is designed to transfer knowledge from one sport to another. Similarly, crossover selection stresses the transfer of content knowledge across different sports. The transfer between sports is specified as “imitative transfer,” “improved transfer,” and “developmental transfer.” This refers to the transfer of one or more training methods from one sport to a crossover sport. Motor skills are a vital part of the content knowledge transfer in crossover athletes’ selection. Motor skills are also the foundation of the training undergone by competitive sports reserves. Besides that, crossover athletes’ selection also involves the transfer from one sport to another in terms of physical education content knowledge, skills, ideas, and consciousness. This is mainly manifested as vertical transfer, that is, the mutual transfer of content knowledge, skills, and concepts between different levels.

A vertical transfer comprises two aspects, namely, upper transfer and lower transfer. The former is an upper concept with higher abstraction levels, while the latter is a lower concept with lower abstraction levels. These superior and inferior concepts are not clearly defined, making it possible for vertical transfer to be bidirectional. On the contrary, horizontal transfer refers to the juxtaposition of content knowledge at the same level. During a knowledge transfer, the human brain assimilates, reconstructs, and integrates the original information. Therefore, standard content knowledge transfer is based on the effect of one kind of learning on another.

To systematically propose KTT, it is necessary for a researcher to scientifically analyze various scenarios in which content knowledge transfer occurs and to explore the laws of transfer performance in various contexts. No matter how it is defined, the goal of any content knowledge transfer is to successfully relocate the source content knowledge to the recipient. It is often difficult to know what type of knowledge can be transferred (Tsang et al., 2004). The cognitive span between transfers based on source content knowledge must not be too large (Tsang, 2002). Scholars have also argued that small gaps in content knowledge may cause recipients to forget old content knowledge before learning a new one. Studies have shown that the nature of the content knowledge transferred, specifically, its reticence and receptivity, has a significant impact on the ease of transfer.

The practical meaning of crossover athletes’ selection is to change the “redundant movements that occur by default in athletes.” Excitatory processes in various parts of the cerebral cortex are widespread in response to a variety of new stimuli, while internal inhibition is relatively backward (Mukminin et al., 2017). The KTT can be bidirectional, positive, and negative. Overcoming negative transfer and conforming to positive transfer is fundamental in crossover selection. By using inspiration reasonably and paying attention to the athletes’ self-learning abilities, training for content knowledge transfer will become a positive transfer (Maijiu et al., 2018).

As a result, the effect of KTT on crossover athletes’ selection involves two sports with similar competitive characteristics, similar levels of competition, similar training processes, and similar evaluation of results. The application of KTT could accelerate the infiltration of the content knowledge system into another skill, that is, the process of theory guiding practice which is defined as “effect.”

A brief review of the literature (Table 2) reveals that some studies show data extraction and quality assessment, with a total score of 1–10. A score of one represents the weakest result and 10 represents the strongest. Table 2 presents a balanced, comprehensive, and critical view of the researched area. Tables 3–5 show the effect of KTT on crossover winter sports athletes’ selection.

**TABLE 2:** Summary of quality assessment scores.

References	Eligibility criteria	Random allocation	Allocation concealment	Group similar at baseline	Blind therapist	Blind assessor	Follow-up	Intention to treat analysis	Between-group comparisons	Point measure and variability	PEDro score
Cummings and Teng (2003)	1	1	1	1	0	0	0	0	1	1	6
Pérez-Nordtvedt et al. (2008)	1	1	1	1	0	0	0	1	1	1	7
Vaeyens et al. (2009)	1	0	0	1	0	0	0	1	0	1	4
Rioux (2011)	1	1	1	1	0	0	0	1	0	1	6
Gulbin et al. (2013)	1	1	1	1	0	1	0	1	1	1	8
Collins et al. (2014)	1	1	1	1	0	0	0	1	1	1	7
Rea and Lavallee (2015)	1	1	0	0	0	0	0	0	0	1	3
MacNamara and Collins (2015)	1	1	0	1	0	0	0	0	1	1	5
Rea and Lavallee (2015)	1	1	0	0	0	0	0	0	1	1	4
Di and Xu (2016)	1	1	0	0	0	0	0	0	0	1	3
Li et al. (2018)	1	1	1	0	0	1	0	0	0	1	5
Li et al. (2018)	1	0	0	0	0	0	0	1	0	1	3
Yang (2021)	1	0	0	0	0	0	0	0	0	1	2
Sun and Gong (2019)	1	1	0	1	0	0	0	1	0	1	5
Gulbin et al. (2013)	1	1	1	1	0	1	0	1	1	1	8
Wenjuan (2021)	1	1	1	1	0	0	0	1	1	1	7
Xia et al. (2021)	1	1	1	1	0	0	0	1	1	1	7

**TABLE 3:** Inclusion criteria according to the PICOS conditions.

Items	Detailed inclusion criteria
Population	Crossover athletes
Intervention	Different sports content
Comparison	Factors of athletes’ skill achievement and knowledge transfer (transfer, psychology, intelligence, and consciousness)
Outcome	Skills performance

**TABLE 4:** Selection of crossover athletes from around the world.

Name	Sport	Achievement	Group	Comparison	Time	Place/Event	Method	Dimension
Eddie Eagan	Lightweight boxing	1 Gold medal	Professional athlete	4-Person skiing	1932	Los Angeles Olympic Games Champion	Individual	Skill
Wen Ying	Track and field	16th Place	Professional athlete	Cross-country skiing	2020	Grova, Norway	Individual	Skill
Zhang Peimeng	Dash	–	Professional athlete	Skeleton	2022	Beijing Winter Olympics	Individual	Skill
Curtis McGrath	Swimming	Gold medal	Professional athlete	Still-water Kayaking	2016	Rio Paralympic Games	Individual	Skill
Angela Hannah	Middle and Long-distance running; Kayaking	5th Place	Professional athlete	Olympic Kayak	2012	London Olympic games	Individual	Skill
Chris Witty	Speed skating	5th Place	Professional athlete	500-m time trial in cycling	2000	Sydney Olympic games	Individual	Skill
Yelena Isinbayeva	Rhythmic gymnastics	Gold medals	Professional athlete	Pole vault	2004, 2008	Olympic games	Individual	Skill
Knut Anders Fostervold	Football	First division	Professional athlete	Road cycling	2019	World championships	Individual	Skill
Ester Ledecka	Pole vault	2 Gold medals	Professional athlete	Alpine Skiing Giant Slalom and Snowboarding Parallel Giant Slalom	2018	Pyeong Chang Winter Olympics	Individual	Skill
Shaun White	Snowboarding on U-shaped Slopes	Gold medal	Professional athlete	Extreme skateboard	2010	Vancouver 2010 winter Olympics	Individual	Skill
Dirk Nowitzki	Tennis	No given	Professional athlete	Basketball	No given	No given	Individual	Skill
Rebecca Romero	Rowing	Gold medal	Professional athlete	Track cycling	2008	Beijing Olympic games	Individual	Skill
Hayley Wichenheiser	Ice-hockey	Runner-up	Professional athlete	Softball	2004	Greek Olympic games	Individual	Skill
Liu Xiang	100-m Sprint	World champions	Professional athlete	Hurdle	2018	Beijing Olympic games	Individual	Skill
Clara Hughes	Road bike	1 Gold medal, 1 silver medal, and 1 bronze medal	Professional athlete	Speed skating shampion	2002–2006	Salt Lake winter Olympics	Individual	Skill
Christa Luding-Rothensburger	Speed skating	1 Silver medal	Professional athlete	10 km Bicycle	1988	Seoul Olympic games	Individual	Skill
Willie Davenport	110 m Hurdles	12th Place	Professional athlete	4-Person snowmobile	1980	Moscow Olympic games	Individual	Skill
Guo Dandan	Gymnastics	World champion	Professional athlete	Free skiing	2021	World cup freestyle skiing series, Australia	Individual	Skill
Jacob Tullin Thams	Ski jumping	Runner-up	Professional athlete	Sailing	1936	Berlin Olympic games	Individual	Skill
Guo Dan	Roller skating	6th Place	Professional athlete	5,000 m speed skating	2016	National winter games	Individual	Skill
Yelena Isinbaeva	Artistic gymnast	Gold medal	Professional athlete	Pole vault	2004, 2008	Greece and Beijing Olympic games	Individual	Skill
Clara Hughes	Cycling	Three additional medals (including a gold)	Professional athlete	Speed skating	2002, 2006	Salt Lake and Torino Olympic games	Individual	Skill
Rebecca Romero	Rower	Gold medal	Professional athlete	Cycling	2008	Beijing Olympic games	Individual	Skill
10 of the 12 finalists in female diving started gymnastics	Gymnastics	Finalists in female diving	Professional athlete	Female diving	2004	Athens Olympic games	Individual	Skill

**TABLE 5:** Selection of crossover Olympic champions.

No.	Name	Gender	Primary age	Content	Final age	Content	Champion age	Height/cm	Weight/kg
1	Gao Ming	Female	4	Swimming	6	Diving	18	163	52
3	Fu Mingxia	Female	5	Gymnastics	6	Diving	14	160	48
5	Sun Mingfu	Female	13	shot	15	Judo	22	178	100
6	Tang Shengling	Male	8	Diving	11	Weightlifting	25	160	60
7	Le Jingyi	Female	7	High jump	9	Swimming	21	175	63
8	Yang Xia	Female	8	Track and field	11	Weightlifting	23	150	53
9	Lin Weining	Female	12	Wushu	13	Weightlifting	21	163	69
10	Li Na	Female	5	Skill	8	Diving	16	162	46
11	Sang Xue	Female	5	Gymnastics	10	Diving	16	158	40
12	Xi Dongmei	Female	13	Wrestling	15	Judo	29	158	52
13	Meng Guanlinag	Male	9	Swimming	17	Canoe and kayak	27	182	82
14	Liu Xiang	Male	7	Long jump	13	Hurdles	21	189	87
15	Wang Xun	Female	8	Judo	13	Wrestling	19	172	75
16	Luo Wei	Female	12	Hurdles	16	Taekwondo	21	180	67
17	Zhao Ruirui	Female	10	Basketball	12	Volleyball	23	197	75
18	Liu Chunhong	Female	8	Judo	11	Weightlifting	19	165	70
19	Lin Yue	Male	5	Gymnastics	7	Diving	17	157	46
20	Qin Kai	Male	4	Gymnastics	8	Diving	22	170	65
21	Zou Shiming	Male	12	Sanda	14	Boxing	27	162	48
22	Yang Xiuli	Female	12	Shot	16	Judo	25	173	77
23	Huo Liang	Male	7	Gymnastics	8	Diving	19	158	50
24	Zhong Man	Male	12	High jump	13	Fencing	25	190	75
25	Wang Feng	Male	5	Gymnastics	8	Diving	29	173	65
26	Jin Ziwei	Female	12	Basketball	14	Rowing	23	184	78
27	Xi Aihua	Female	12	Shot	15	Rowing	26	182	85
28	Zhang Yangyang	Female	11	Basketball	15	Rowing	19	185	83
29	He Wenna	Female	6	Gymnastics	9	10–a trampoline	19	160	48
30	Dun Dong	Male	7	Gymnastics	13	Trampoline	23	168	56
31	Lu Chunlong	Male	5	Gymnastics	9	Trampoline	19	170	57
32	Yi Sinlin	Female	12	Track and field	13	Shooting	23	165	51
33	Zhang Yanquan	Male	4	Gymnastics	10	Diving	18	158	52
34	Wang Hao	Female	4	Gymnastics	9	Diving	20	156	50
35	Lin Qingfeng	Male	9	Shot	11	weightlifting	23	167	70
36	Dong Dun	Male	6	Gymnastics	13	Trampoline	23	168	56
37	Xu Anqi	Female	10	Basketball	12	Fencing	20	183	75

### Population

The athletes are not only limited to those from China but also from different countries around the world. KTT focuses on the technical achievements of national professional athletes and mainly studies the improvement of the skill achievements of professional athletes. For example, twenty-six outstanding scholarship winners have reached the elite level of competition in the past (Gulbin et al., 2013). The standard of cross-sport is to reach the level of the provincial team in both pre-cross sports and post-cross sports. For example, the training targets include all high-level athletes of national standards. Moreover, out of the seven contestants, five of them won international medals (MacNamara and Collins, 2015). In a study by Yanchun et al. (2021), 59 outstanding cross-country skiers form the national cross-country ski team. Moreover, Great Britain, Australia, Canada, and the USA 2012 and 2010 Olympic teams (*n* = 2,307) were reviewed (Collins et al., 2014; Tai et al., 2020; Hao, 2020).

Notably, the review found that there are more women than men, with the ratio of females: To males is 21:15. The training cycle is a minimum of 1 year and a maximum of 7 years (Wenjuan, 2021). In addition, in the study of Collins et al. (2014), TT occurred most regularly between the ages of 11 and 32 years.

Collins et al. (2014) mentioned that in the 2010–2012 Olympic Games, the types of selection of crossover winter sports athletes, including organized and inorganized. Rea and Lavallee (2015) demonstrated the selection of crossover winter sports athletes type: skills-dominant and physical-dominant types. The skills-dominant type is the athletes’ age range of 12.67 ± 0.58 years, while the physical fitness-dominant type is the athletes’ age range of 10.13 ±1.13 years.

### Intervention

Based on the included review, the following intervention characteristics were reported: (1) Sports events using CSM, (2) types of cross-event selection, and (3) intervention measures. Details of the intervention are as follows:

(1) Review of CSM for winter sports shows that they concentrate mainly on exercise skills. CSM of games in Winter Olympics: freestyle skiing, snowboarding, speed skating, short track speed skating, figure skating, cross-country skiing, alpine skiing, ice hockey, snowmobile, steel frame snowmobile, and bobsled. Freestyle skiing includes aerial, U-shaped field, and slope obstacle skills (Gulbin, 2008; Vaeyens et al., 2009; Farrow et al., 2013). Other sports events include swimming, shooting, diving, high jump, track and field, Wushu, skill type, wrestling, long jump, hurdles, basketball, judo, Sanda, and gymnastics (Wenjuan, 2021). Additionally, crossover athletes for snowboarding include those originally from gymnastics, diving, trampoline, acrobatics, and dance. Meanwhile, crossover athletes for speed skating are from gymnastics, diving, trampoline, and dance. (2) Types of crossover athletes’ selection. Gulbin et al. (2013) showed that there are three types of cross-event selection: cross-events of similar events (such as volleyball to sand volleyball), similar events (such as gymnastics to water jump), and dissimilar events/requirements (such as boxing to sailing). (3) Vaeyens et al. (2009) have given specific intervention measures. In the first stage, they make a recruitment call to qualified UK sports through the mass media. In the second stage, athletes attended the assessment events. In the third stage, they specified training interventions (Vaeyens et al., 2009).

### Comparison

Based on the effect of KTT on the selection of crossover winter sports athletes, the CSM used in the reviewed literature was compared. The review found that the skill performance of athletes before and after crossover was assessed using the following methods: (i) Using a non-experimental descriptive cross-sectional qualitative and descriptive method. A comparative study that replied to the investigation interviews and observation was used to study the transfer of talents (Collins et al., 2014); (ii) Using the phenomenological method as a qualitative method (Rea and Lavallee, 2015); (iii) A semi-structured interview methodology (MacNamara and Collins, 2015); and (iv) Quantitative examination (Yanli, 2020). All the training methods used are discrimination training methods. According to the uniqueness of KTT, only specific training methods were adopted by the researcher.

Notably, for a comprehensive review, the researchers did not just take a representative sample but did an objective survey. For example, based on Collins et al.’s (2014) research on successful talent transfer, during the review process, interviews and observation methods need to be emphasized, which extends previous investigations found in the literature and opens some possibilities to improve the theory.

Collins et al. (2014) studied the effectiveness of KTT on the selection of crossover winter sports athletes TT through a two-part study:

Stage 1—Epidemiology of talent transfer: (1) Design: Employ an epidemiological approach to gain information regarding the characteristics of completed TT. (2) Procedure: In total, 204 nations competed. Countries were purposefully sampled for their proactive involvement and utilization of TT. (3) Participants: Athlete profiles (n = 2,307) from the four sampled countries. (4) Data analysis: First, the occurrence of TT was examined (i.e., how many times it happened and in which sports); second, this information was then analyzed for patterns and to decipher the most common donor and transfer sports; and third, demographic information was analyzed (i.e., age of TT).

Stage 2—A qualitative exploration of talent transfer: (1) Design: To examine how the scientific logic of TT stands up to scrutiny by discipline specialists, a qualitative methodology was undertaken using semi-structured interviews (Kallio et al., 2016). (2) Participants: Four participants participated (Kallio et al., 2016). (3) Procedure: The interviews were 40–55 min (Bolderston, 2012) where participants were provided with 30 examples of TT taken (i.e., sprinting to bobsleigh) where they were asked to score. (4) Data analysis: Interviews were transcribed verbatim, statistical participants’ scores of transfer factors, using an interpretive description approach (Thorne et al., 1997). (5) Analysis reviewing the comments, categorizing key themes, and confirmation phase (Maheu and Thorne, 2008).

### Outcome

The outcome includes effectiveness and lacks evidence-based direction and a rigorous exploration and support mechanism (MacNamara and Collins, 2015). The specific outcomes are as follows:

First, *effectiveness*. The outcomes focus on the technical achievements of national professional athletes and primarily study the improvement of the skill achievements of professional athletes. Skill achievements mainly focus on the skills-dominant and physical-dominant types (Wenjuan, 2021). Furthermore, Tables 3, 4 and supports the possibility of the Selection of Crossover Winter Sports Athletics. Elements that contribute to knowledge transfer are the individual’s level of mastery, sports games experiences, and educational and work experiences (Rioux, 2011).

The personal factors that support talent transfer are previous sports experience, physiological characteristics of athletes, and psychological and behavioral factors of athletes (MacNamara, 2045). R577X polymorphism of the ACTN3 gene can be used as the indicator for athlete selection, where people with the X allele or RX genotype are more suitable for cross-country skiing (Li et al., 2018). Talent crossover athletes can move from one sport to another and get into the elite level of another with significant results (Gulbin et al., 2013; Vaeyens et al., 2009). In the UK, skills, physics, physiology, perception, and cognition are the main indicators of the selection of crossover winter sports athletes (Vaeyens et al., 2009). Vaeyens et al. (2009) showed that they will be able to transfer the sports experience, physiological ability, and skills from the previous events to the post-event and achieve rapid success in the former. Bullock et al. (2009) showed that the existing high-level athletes have changed from one sport to another. By minimizing the problems of adolescent development, shortening the training period of athletes, and maximizing the return of those older athletes who have invested in the training process, the ability of cross-athletes (in post-cross events) to reach a high level of adulthood can be improved.

According to Xia et al. (2021), proper research should start by exploring the specific transfer theory on the crossover selection of athletes. This should be followed by exploring the connection between old and new knowledge of sports before and after the crossover selection. Finally, consideration should be made of the common elements between the sports before and after the crossover. The common elements for knowledge transfer of crossover selection are the specific and abstract mutual match that forms the primary method for knowledge transfer. Some transfers take place which does have obvious physiological or motor skills crossover (Xia et al., 2021).

Second, *scholars’ views are often not consistent*. Some scholars are of the view that not all sports can be transferred (Vaeyens et al., 2009). KTT lacks evidence-based direction as well as a rigorous exploration and support mechanism for this method in improving skill achievement (MacNamara and Collins, 2015). *The outcomes are as follows. (i) Effectiveness—The publicized answer: TT would seem to be effective but perhaps not solely as a formal initiative. The information shows that TT occurred most regularly between the ages of 11 and 32 years. (ii) Mechanisms of selection and investment—Expert opinion: The ability to learn new skills was perceived as more important than the possession of transferable skills. Physical mechanisms were associated with successful TT. However, researchers suggested that it may not be the most important factor. Finally, athletes’ previous experience in sports emerged as a possible mechanism for success.* The most widely supported factor was an athlete’s ability to push themselves beyond their limits.

## Discussion

### Discussion on theoretical system for KTT and crossover athletes selection

*Based on a review of KTT and crossover athletes selection literature, the researcher discusses the following:*

By improving thinking skills and mastering positive transfer methods, athletes would be able to complete the transfer (Vaeyens et al., 2009). When athletes master the skills through training, their cortical cognition also undergoes dynamic changes, confirming the traces and adaptability of cognitive processing in skill learning and ensuring the success of crossover selection (Zhang et al., 2006). By acquiring knowledge of exercise physiology, athletes have a positive transfer to master physical exercise principles (Rea and Lavallee, 2017). For instance, when transferring previously learned technical movements to crossover sports for further learning, coaches should make full use of athletes’ existing knowledge and skills and inspire them to use existing knowledge to cultivate their methods of applying technical principles (Wenjuan, 2021). Additionally, there is a need to characterize the diversity of learning situations. Having good knowledge of crossover sports events, designing the scenes of intentional learning, and guiding the discovery of learning in training help strengthen the strategy of training for specific crossover sports (Li et al., 2018). Accordingly, it is necessary to emphasize principles, master basic technical movements, highlight internal connections between crossover sports events, and create suitable conditions for the knowledge transfer of crossover selection (Wang et al., 2019). Therefore, the *crossover athletes’ selection* should be based on KTT, which can guide crossover athletes’ selection. Meanwhile, crossover athletes must follow the basic principles of KTT and its physical and psychological characteristics (Xia et al., 2021). The goal is to highlight the concept of basic technical actions and ensure that the generalization of the ability of athletes has been achieved.

There must be common factors among the various sports to be transferred. These common factors represent the KTT and crossover selection on specific contents, and they form a one-to-one community (Cummings and Teng, 2003). Given this, there must be an appropriate commonality between crossover sports. It is essential to focus on strengthening the determinants of the common factors for transferring knowledge (Vaeyens et al., 2009). Furthermore, there must be a generalized level of existing sports skills and experience (Gulbin et al., 2013). As for the characteristics between the new and old sports knowledge and skills, the more common the factors are, the easier it is to realize crossover knowledge transfer (Sun and Gong, 2019). Previous learning provides preparation for subsequent learning, whereas subsequent learning promotes progress and depth of prior learning (Gulbin et al., 2013). Furthermore, the more precise the regulations and the clearer the hierarchy, the easier it is to realize the crossover transfer of sports knowledge. The stronger the athletes’ original sports skills foundation, knowledge, and experience, the higher the discipline to achieve better results in crossover sports (Di and Xu, 2016; Sun and Gong, 2019).

### Discussion on the effect of KTT on crossover athletes’ selection

Crossover selection can achieve its goal by broadening sources (Chenguang and Menghan, 2022). The selection should focus on speed, endurance, and strength for physical-oriented events. Meanwhile, skills-oriented events should select athletes from challenging sports (Lei, 1997). Skills-oriented and psychological athletes should be selected from sports of accuracy. Meanwhile, skills-oriented physiological athletes should be selected from sports with network confrontation, usual confrontation, and rotation defense (Bullock et al., 2009). It should be noted, however, that athletes must be able to self-analyze crossover selection, as their cognitive structure affects their crossover selection (Wu, 2020).

There are effects of KTT on crossover athletes’ selection for other sports. For example, the crossover of track and field sports to winter sports has significant advantages (Gao et al., 2018). Among them is the fact that it is a perfect match where special training for crossover winter sports athletes can be reduced as much as possible (Ma and Wang, 2020). Therefore, the successful utilization of game tactics and competition awareness requires a certain degree of movement skills, and the successful execution of movement skills requires certain physical conditions (Maijiu et al., 2018). Moreover, higher athletes’ intelligence, including emotional intelligence, leads to a quick transfer of crossover knowledge (Li et al., 2018). Training crossover athletes makes it easier for them to succeed in the same sports group (Baker et al., 2002; Gao et al., 2018; Ma and Wang, 2020; Rilang, 2020).

### Limitations

There are limitations in the literature search (i.e., there are more CNKI database studies to be reviewed). However, in searching the literature, based on the inclusion and exclusion criteria of PRISMA, the deviation was reduced and ensured the objectivity of the literature review.

Some limitations of the review are as follows: First is the sample limitation, where the review only focuses on the following geographies: Australia, the United Kingdom, Australia, USA, Russia, and China (Vaeyens et al., 2009; Di and Xu, 2016; Li et al., 2018; Qunru, 2019; Sun and Gong, 2019; Xia et al., 2021; Yanchun et al., 2021; Yang, 2021), and Canada (Collins et al., 2014). Second is the review’s small sample size. Even so, it is consistent with the results of many KTT research studies (i.e., Szulanski, 1996; Lane and Lubatkin, 1998; Tyler and Steensma, 1998; Bresman et al., 1999; Cummings and Teng, 2003; Xiaohua and Zhejian, 2020).

## Conclusion

This systematic review aims to make up for the shortcomings of the current crossover selection work and promote the improvement of the athletes’ crossover selection system. Furthermore, this review also aspires to improve the theoretical system of crossover selection and the management system of crossover selection. In addition, this review promotes cross-sports integration.

Concerning the ease of transfer, athletes with more KTT experience find it easier for their knowledge to be transferred to other sports. KTT has beneficial effects on the selection of crossover winter sports athletes. Furthermore, the researchers focus on physical and technical factors such as anthropometrics, psychology, physiology, or required motor skills, which seem to be considered central to the selection process. Nevertheless, physical mechanisms were a point of contention for the participants. However, scholars have suggested that it may not be the most important factor. In the meantime, it appeared that the ability to learn new skills was perceived as more important than the possession of transferable skills.

### Future research and recommendations

#### Future research

Future research should involve in-depth analyses of the comprehensive factors that may affect athletes’ crossover and KTT. Inspired by the interview and observations method of Collins et al. (2014), future research may do more psychological analysis based on observations of athletes’ and coaches’ screening and/or behavior.

#### Recommendations

Strengthening athletes’ psychological and KTT training from the psychological perspective is imperative to actualize the comprehensive factors influencing knowledge transfer and learning principles. Another aspect to explore is the development or improvement of winter sports’ reserve athletes by solving problems of unsteady crossover athletes selection and low adaption levels.

The promotion of public winter sports suggests innovative physical mechanisms. Regarding crossover content, similar textbooks should be developed for athletes to understand their common factors. In addition, future research may provide more insights into whether the successful transformation applies to all forms of sports or only to specific categories.

The relevant authorities should open some possibilities to increase the application of KTT in the selection of crossover. This could be done through the application of transfer theory on the selection of crossover to physical education in general sports. Moreover, universities can expand their athlete’s skills for crossover sports. They may also consider restructuring current KTT programs to be more focused on psychological screening and interviews/behavioral observation.

## Data availability statement

The original contributions presented in this study are included in the article/supplementary material, further inquiries can be directed to the corresponding author.

## Author contributions

All authors listed have made a substantial, direct, and intellectual contribution to the work, and approved it for publication.
